# SARS-CoV-2 Evasion of the Interferon System: Can We Restore Its Effectiveness?

**DOI:** 10.3390/ijms24119353

**Published:** 2023-05-27

**Authors:** Alessandra Sacchi, Flavia Giannessi, Andrea Sabatini, Zulema Antonia Percario, Elisabetta Affabris

**Affiliations:** Laboratory of Molecular Virology and Antimicrobial Immunity, Department of Science, Roma Tre University, 00146 Rome, Italy; alessandra.sacchi@uniroma3.it (A.S.); flavia.giannessi@uniroma3.it (F.G.); andrea.sabatini@uniroma3.it (A.S.); zulema.percario@uniroma3.it (Z.A.P.)

**Keywords:** type I interferons, type III interferons, SARS-CoV-2, RNA viruses, innate immune system evasion

## Abstract

Type I and III Interferons (IFNs) are the first lines of defense in microbial infections. They critically block early animal virus infection, replication, spread, and tropism to promote the adaptive immune response. Type I IFNs induce a systemic response that impacts nearly every cell in the host, while type III IFNs’ susceptibility is restricted to anatomic barriers and selected immune cells. Both IFN types are critical cytokines for the antiviral response against epithelium-tropic viruses being effectors of innate immunity and regulators of the development of the adaptive immune response. Indeed, the innate antiviral immune response is essential to limit virus replication at the early stages of infection, thus reducing viral spread and pathogenesis. However, many animal viruses have evolved strategies to evade the antiviral immune response. The *Coronaviridae* are viruses with the largest genome among the RNA viruses. *Severe Acute Respiratory Syndrome-Coronavirus*-*2* (SARS-CoV-2) caused the coronavirus disease 2019 (COVID-19) pandemic. The virus has evolved numerous strategies to contrast the IFN system immunity. We intend to describe the virus-mediated evasion of the IFN responses by going through the main phases: First, the molecular mechanisms involved; second, the role of the genetic background of IFN production during SARS-CoV-2 infection; and third, the potential novel approaches to contrast viral pathogenesis by restoring endogenous type I and III IFNs production and sensitivity at the sites of infection.

## 1. Introduction

An acute respiratory syndrome outbreak of unknown etiology was reported in Wuhan, China [1] in December 2019. The virus that caused the disease was isolated and its genome was sequenced soon after. It is a new coronavirus named *Severe Acute Respiratory Syndrome Coronavirus* type *2* (SARS-CoV-2) based on its similarity to SARS-CoV-1 and *Middle Eastern Respiratory Syndrome-Coronavirus* (MERS-CoV), the two new human coronaviruses causing severe respiratory syndrome epidemics in 2003 and 2012, respectively. SARS-CoV-2 primarily causes infection in the respiratory tract, showing a broad spectrum of clinical patterns named coronavirus-induced disease 2019 (COVID-19). The virus infection has caused either an asymptomatic disease in some individuals or a mild to severe illness in other patients. Although the common symptoms of a mild form of the disease have been fever, cough, headache, and myalgia, critically ill patients have suffered from severe pneumonia with acute respiratory distress syndrome (ARDS) and have required intensive care and mechanical ventilation [2].

In March 2020, while COVID-19 spread, the World Health Organization (WHO) declared the COVID-19 outbreak a global pandemic that has caused over 650 million confirmed cases of COVID-19 worldwide up to now, including more than 6.5 million deaths (data from WHO, Weekly Epidemiological Update on COVID-19—21 December 2022). The disease also caused an enormous social and economic impact worldwide [3]. During the last three years, COVID-19 also influenced the scientific community, which still looks for novel strategies to contrast the virus diffusion by developing vaccines and antiviral strategies. The effort led to new vaccine strategies to reduce the pathogenesis of COVID-19 without efficiently eradicating the infection and virus circulation and requested the validation of several antiviral molecules.

Coronaviruses are RNA viruses with the largest known genome (about 30 kDa) and have a limited number of structural proteins (i.e., S, M, E, N, and, in some members of this virus family, HE). They codify many multifunctional viral elements inside the infected cells, recently defined as a viral arsenal [4], and can set up an efficient viral replication together with multiple immune evasion strategies. Furthermore, RNA viruses are well known to mutate highly, and the mutation rate facilitates the development of new variants escaping neutralizing antibody recognition. Today, scientists are far from a detailed knowledge of the virus-host interactions that evolved in the *Coronaviridae* family members. Nonetheless, during the last three years, intensive basic and applied research in the virology field revealed that many viral proteins disrupt multiple steps of the interferon (IFN) system, including the cellular detection of viral RNA, the transmission of the alert signal to the nucleus that activates the synthesis of type I and III IFNs, and the activation and action of IFN-stimulated genes (ISG). Vanderheiden et al. [5] underlined the main role of type I and III IFNs in inhibiting viral replication, at least in vitro. Their findings suggested the need for novel pharmacological strategies to restore the IFN system’s functionality at the infection site to inhibit SARS-CoV-2 replication. Since coronavirus proteins inhibit the IFN system both in the early and late phases of viral expression, it is possible to presume the importance of IFNs in defeating the SARS-CoV-2 infection.

The overall aim of this review is to summarize and update significant findings of the studies on SARS-CoV-2–IFN interactions to identify potential therapeutic targets to restore the physiological IFN induction and control of the SARS-CoV-2 infected cells.

## 2. The IFN System and Its Relevance to Contrast Microbial Infections

IFNs are fundamental effectors of antimicrobial and antitumor innate immunity and important regulators of the adaptive immune response. The first discovery by Isaacs and Lindemann takes us back to 1957. IFNs act in a species-specific manner, and today, we divide them into three antigenically unrelated groups: type I, type II, and type III. Type I IFN is a group of structurally similar cytokines codified by a multi-gene family including 13–14 IFN-α types along with IFN-β, IFN-ε, IFN-κ, IFN-ω, IFN-δ, IFN-ζ, and IFN-τ. IFN-γ is the only member of the type II IFN [6]. Type III IFN, discovered in 2003, contains four members in humans: IFN-λ1/IL-29, IFN-λ2/IL-28A, IFN-λ3/IL-28B, and IFN-λ4 [6,7,8,9]. A restricted number of immune cells can produce IFN-γ (mainly Th1 and NK cells) through antigenic or mitogenic stimuli. Instead, all nucleated cells produce type I and III IFNs by sensing pathogen-associated molecular patterns (PAMPs) or damage-associated molecular patterns (DAMPs). IFN-β gene transcription requires the activation of the constitutive transcription factor IRF3, whereas IFN-α and IFN-λ gene transcription requires the activation of both IRF3 and IRF7. IFN-β is induced rapidly after sensing PAMPs (a couple of hours) via the activation of the constitutively expressed IRF-3 (immediate early expression). If the stimulus persists, it is rapidly followed by IFN-α production (delayed-type expression) because the latter requires the induction of IRF-7 via IFN-β action. Activated IRF3 and IRF7 also maintain IFN-β expression [10,11,12,13]. Regarding IFN-λ, data suggest that the IFN-λ1 gene is regulated by virus-activated IRF3 and IRF7, thus resembling that of the IFN-β gene, whereas IFN-λ2/3 gene expression is mainly controlled by IRF7, thus resembling those of IFN-α genes [12]. Type I IFNs are induced and resolved rapidly, followed by a delayed but sustained induction of IFN-λ genes.

**Figure 1:**
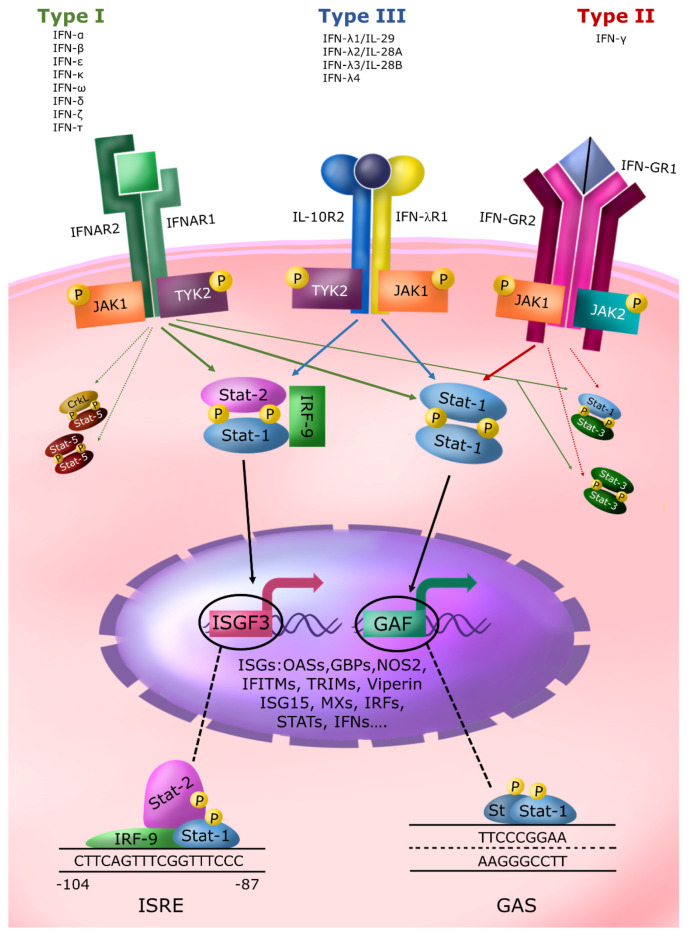
Schematic representation of IFN receptors and signal transduction pathways. See the text for details.

The three IFN types bind to three different specific receptors and use the JAK-STAT signaling as the primary signal transduction pathway (Figure 1). IFNs induce common and type-specific effects with intersections of the signal transduction pathways, depending on the cell type and the environment of different tissues and organs [14,15].

Type I IFN members bind the same cell membrane receptor (IFNAR), consisting of two subunits (IFNAR1 and IFNAR2) and activate a spectrum of activities [16]. Type II IFN (IFN-γ) signals through a receptor (IFNGR) composed of two subunits (IFN-GR1 and IFN-GR2), and all the members of type III IFN share the same receptor complex (IFNLR) composed of the IFN-λ specific subunit IFN-λR1 (IL-28R1) and a subunit in joint with the IL10 receptor (IL-10R2). The receptors for type I and II IFNs and the IL10R2 receptor subunit are widely distributed on the surface of most cell types with few exceptions. On the contrary, the cell surface expression of the high-affinity receptor subunit IFNLR1 is restricted, limiting cell responsiveness to these cytokines mainly to the cells expressing this subunit, such as epithelial cells, liver cells, and subsets of myeloid cells and neuronal cells. This limitation likely explains the importance of IFNλ at the mucosal entry sites and the blood/brain barrier [14,15,16,17].

After the IFNAR-1 and IFNAR-2 subunit interactions due to their recruitment by type I IFN binding, two intracytoplasmic receptor-associated JAK kinases (i.e., Tyk-2, associated with IFNAR-1, and JAK-1, associated with IFNAR-2) become activated with subsequent tyrosine phosphorylation of the intracellular domains of the receptor itself. After phosphorylation, the intracellular domains of the IFN receptor become docking elements for Src homology 2 (SH2) domain.

The phosphotyrosine-binding domain-containing proteins present in the membrane or cytoplasmic compartment are named Signal Transducers and Activators of Transcription proteins (STATs). These are a family of latent cytoplasmic transcription factors that mediate several biological processes, including cell growth, differentiation, apoptosis, fetal development, transformation, inflammation, and immune response. The JAKs phosphorylate the receptor-recruited STATs on a single conserved tyrosine residue in their carboxyl-terminal portion. The tyrosine phosphorylated STATs are released from the cytoplasmic region of the receptor subunits to form homodimers or heterodimers through reciprocal interaction between the phosphotyrosine of one STAT and the SH2 domain of another. Following dimerization, STATs rapidly translocate to the nucleus and interact with specific DNA regulatory elements to induce target genes transcription. Serine phosphorylation on conserved residues increases their transcriptional activity [18].

Since the discovery of STATs and JAKs proteins in the IFN signaling pathways in the early 1990s, several cytokines and growth factors have been recognized to activate various STAT proteins [19,20]. Seven members of the STAT family have been identified in mammalian cells: STAT-1, STAT-2, STAT-3, STAT-4, STAT-5a, STAT-5b, and STAT-6 [21]. Furthermore, convincing evidence from genetic mapping studies indicates a common ancestral origin that gave rise to three chromosomal clusters of STAT genes through a series of duplication processes [22]. The seven existing STATs regulate, in a combinatorial mechanism, the signal transduction pathways of many cytokines and growth factors. Interestingly, type I and III IFNs, via the interaction of their specific membrane receptor, classically activate the same JAK kinase (Tyk-2 and JAK1) and the same STAT proteins (STAT-1 and -2).

Consequently, type III IFN has largely overlapping expression and function with type I IFN; however, some differences exist [23,24]. In addition, type I and III IFN are the only known cytokines that activate STAT-2. STAT-1 and STAT-2 form a heterodimer that associates with another cytoplasmic protein (p48/IRF-9), resulting in the formation of the mature three-molecular complex named IFN-stimulated gene factor 3 (ISGF3). ISGF3 translocates to the nucleus and binds to IFN-stimulated response elements (ISRE) present in the promoter of type I and III IFN-induced genes to activate their transcription (Figure 1). In addition, part of the activated STAT-1 forms another active transcription factor, the STAT-1 homodimers, named GAF (Gamma-activated factor). This name derives from the discovery that IFN-γ leads to a marked STAT-1 tyrosine phosphorylation by JAK-1 and JAK 2 tyrosine kinases through the recruitment of its receptor subunits and, consequently, to the formation mainly of STAT-1 homodimers. The GAF factor binds to its enhancer element named GAS (Gamma Activated Sequence), a nine-nucleotide quasi-palindromic sequence present in the IFN-γ-induced immediate early genes. Type I IFN has been also reported to induce sometimes the tyrosine phosphorylation of other STATs (i.e., STAT-3 or STAT-5) in a cell-dependent manner causing the formation of other transcription factors of the STAT family, i.e., STAT 3:3 homodimers, STAT 1:3 heterodimers, STAT 5:5 homodimers, and CrkL: STAT-5 heterodimers that bind GAS-like element. IFN-γ activates STAT-3, but less efficiently than STAT-1, thus inducing the formation of a certain level of STAT 3:3 homodimers and STAT 1:3 heterodimers.

In conclusion, the three IFN types use different cell membrane receptors, holding some common signal transduction elements and different levels of overlapping effects. Studies in STAT-1 and STAT-2 knockout mice [25,26] have unequivocally demonstrated their central role in the biological activities of the different IFN types. IFNs can also activate the PI3K/AKT/mTOR pathway and other, less characterized signaling tuning the capacity of the JAK/STAT signal transduction pathway to diversify their biological response.

IFNs regulate hundreds of genes through the activation of the JAK-STAT signal transduction pathway. A valuable online open-access database (Interferome, version 2.01, http://interferome.its.monash.edu.au/interferome/home.jspx, accessed on January 2013) enables a comprehensive vision of IFN-regulated processes and pathways by a compilation of microarray datasets derived from various cell types and tissues stimulated with type I, II, and III IFNs [27,28,29]. The overall IFN-induced effect is complex and, in part, cell-type dependent. Among the induced genes, named ISG (IFN-stimulated genes), there are members of the transcription factor family Interferon Response Factors (IRF), including IRF1, IRF2, IRF3, and IRF7 [30], which bind specific ISRE-like enhancers, and ISRE. Therefore, after the «immediate early» transcriptional induction of ISG due to ISGF3 and/or GAF formation, there is also the cycloheximide-dependent «immediate late» transcriptional regulation due to IRF1, IRF3, and IRF-7 production and their gene expression regulation [10]. IRF1 has tumor suppressor [31,32] and antimicrobial functions [33], whereas IRF7 is required for the activation of the IFN-α and -λ gene transcription that, as mentioned before, requires the IRF3-IRF7 activated heterodimers.

Regarding antimicrobial ISG, they can be subdivided into effectors and regulatory genes. Effectors genes codify restriction factors that act directly targeting pathogens, examples are 2′-5′ oligo-A synthetase (OAS), which induces viral RNA degradation; Protein Kinase R (PKR) acts by inhibiting viral protein synthesis; Promyelocytic leukemia (PML) protein, the component of PML Nuclear Bodies involved in DNA viruses and retrovirus restriction [34]; Tetherin/BST-2 inhibiting envelope virus release; ISG-15, a ubiquitin-like modifier induced by type I and III IFN, that can be also secreted by the cell and binds the LFA-1 integrin receptor on NK- and T-cells to potentiate their production of type II IFN [35]. OAS and PKR are allosteric enzymes that require activation by dsRNA detection. Moreover, ISG15, in a ubiquitin-like fashion, is covalently linked by its C-terminal LRLRGG motif to lysine residues on newly synthesized proteins, a process termed ISGylation. The effect of ISGylation is incompletely understood and involves both activation and inhibition of antiviral immunity. Other restriction factors are APOBEC3G cytidine deaminase [36] and ADAR-1 p110 isoform, a dsRNA editing enzyme, that increase the rate of RNA virus mutation, and iNOS, which mediate the production of high concentration of NO. The regulatory genes codify factors modulate innate and adaptive immune response; examples are nucleic acid sensors, MHC-class I and II antigens; Fc receptors; IRFs; and STAT-1 and 2 themselves.

Type I and III IFNs are involved in the multi-level regulation of antiviral and antitumor innate responses. In contrast, type II IFN is more linked to activation and perpetuation of inflammation, and to adaptive cell-mediated response. Type I IFNs induce differentiation, maturation, and activation of myeloid dendritic cells (mDCs) that, in turn, promote antiviral T-cell immunity [37]. Moreover, the type I IFN released by plasmacytoid dendritic cells (pDCs), a dendritic cell subset specialized in producing a high level of IFN-α, stimulates the activation of NK cells, biases CD4 T cells toward a Th1 response, enhances CD8+ T cell functions, induces memory CD8+ T cells, and promotes the development of regulatory T (Tregs) cells and the differentiation of B cells into antibody-secreting plasma cells [38]. Type I IFN also possesses inflammatory properties through the activation of NLRP3 inflammasome that leads to the production of IL-1β and IL-18 and finally to pro-inflammatory cell death, named pyroptosis (reviewed in [38]). Like other multifunctional cytokines, their excessive or inappropriate activity can cause toxicity, and even death [39,40,41,42]. In this respect, an IFN signature, i.e., increased or constitutive production of IFN-α and ISG, has been observed in different human pathologies. An emblematic example is the HIV infection, where IFN-α is chronically produced and appears to be a double-edged sword. Although it exerts potent antiviral properties by reducing viral replication and inducing apoptosis in HIV-infected cells, both human and animal studies support the role of IFN-α in the immune activation and inflammation during HIV chronic infection [43,44,45]. Kinetics of the early innate immune activation during HIV-1 infection of humanized mice have further shed light on the complexity of the interaction with the IFN system during the infection’s eclipse, burst, and chronic phase [46]. More details about the IFN system are available from others [14,17,24,47,48,49,50,51,52,53].

## 3. Coronavirus Replication

**Figure 2:**
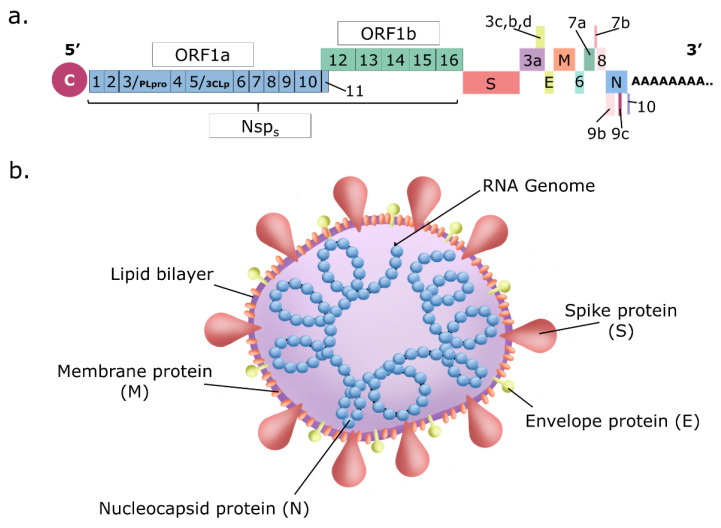
SARS-CoV-2 biology: schematic representation of single-stranded, positive-sense RNA genome (**a**) and viral particle (**b**) of SARS-CoV-2 (See Section 3 for details).

The members of the *Coronaviridae* family are enveloped lytic viruses with unsegmented, single-stranded, positive-sense RNA genomes enclosed by a 5′-cap and 3′-poly(A) tail (Figure 2a). These viruses have the longest known viral RNA genome (about 30 KDa in length). It codifies very few viral structural proteins (i.e., 4 or 5): S, which is cleaved in S1 + S2 and forms the surface trimeric viral receptor, M, the membrane protein, E, the envelope protein, N, the nucleocapsid protein, and HE, the esterase, presents only in some members and not in SARS-CoV-2. In contrast, several non-structural and accessory proteins are produced by coronaviruses inside the infected cells. The viral particles are spherical or pleomorphic in shape, with a diameter of about 60–140 nm (Figure 2b). Single virions are encircled with an envelope containing the viral ribonucleocapsid complex formed by the genome and the nucleocapsid protein N surrounding it. In the envelope are embedded the membrane protein M, a tetraspanin, and the envelope protein E (plus the esterase HE in some family members). Inserted in and protruding from the envelope is the S2 subunit of the viral receptor that is associated with the S1 subunit forming distinct trimeric spikes of 9–12 nm on the surface and giving the virus the appearance of solar corona when observed by electron microscopy. Many general reviews (i.e., ref. [54,55,56,57]) textbook chapters (i.e., ref. [58,59,60]), and a useful website (SARS-Cov-2 resource ~ ViralZone (https//viralzoneexpasy.org/9056, accessed on January 2011) on these viruses and their replication are available. However, the detailed knowledge of the function of all the viral proteins in the viral life cycle is still far away.

**Figure 3:**
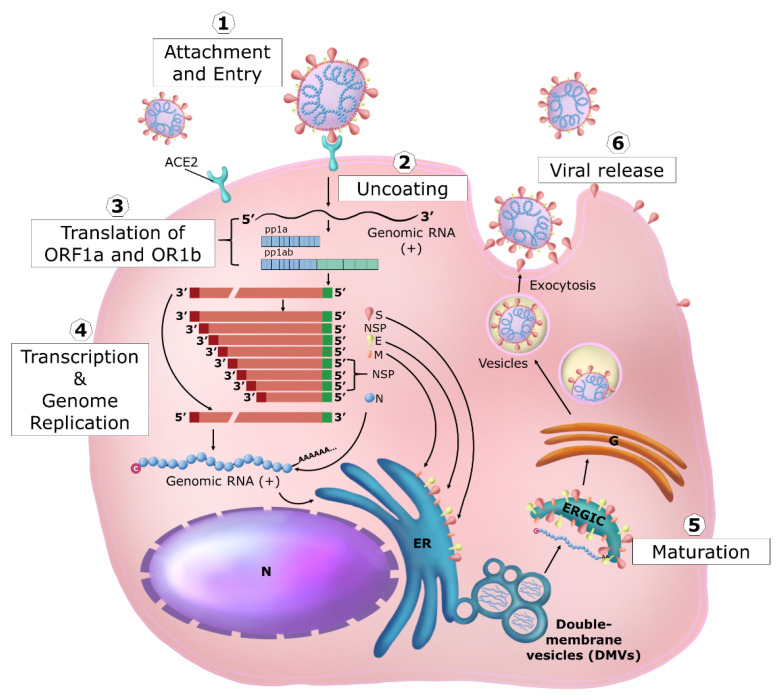
SARS-CoV-2 biology: schematic representation of SARS-CoV-2 replication cycle (see Section 3 for details).

SARS-CoV-2 genome has 79% identity with SARS-CoV and 50% with MERS-CoV, the other two coronaviruses that cause severe respiratory syndromes [61,62]. SARS-CoV-2 belongs taxonomically to the *Betacoronavirus* genus and is a member of the *Coronavirinae* subfamily, *Nidovirales* order, and *Riboviria* realm [63,64]. The viral life cycle is briefly summarized here below (see Figure 3 for a schematic representation).

SARS-CoV-2 S1 subunit engages angiotensin-converting enzyme 2 (ACE2) as the entry receptor and employs the cellular serine protease TMPRSS2 for S2 protein priming required for fusion [65]. Both cellular proteins are expressed in epithelial lung cells, the main target of infection (e.g., ciliated and goblet cells in the airways and cell subset in the lung named surfactant producing type 2 alveolar cells/type II pneumocytes). Since a proportion of patients showed extrapulmonary symptoms, SARS-CoV-2 can likely infect a wide range of cells expressing ACE2 from other organs, such as the heart, kidney, testis, eye, endothelium, and intestinal epithelium [66]. However, other mechanisms of SARS-CoV-2 cell entry can occur that involve other receptors such as the extracellular domain of broadly expressed integrin α5β1 showing an affinity comparable to that of SARS-CoV-2 for ACE2 [67,68]. Unlike epithelial cells, immune cells do not express ACE2 or TMPRSS2, while they do express CD147, described as another receptor for SARS-CoV-2, thus providing an additional route for viral entry [69]. Furthermore, neuropilin-1 has been described as a facilitator of SARS-CoV-2 cell entry and infectivity [70].

After absorption, the virus enters the cell by membrane fusion mediated by the maturation of the C-terminal subunit S2 and consequent exposure of the fusion peptide, releasing the helical ribonucleoprotein complex in the cytoplasm.

Once in the cytoplasm, the ribonucleoprotein complex immediately starts genome expression by translation of about two-thirds of the 5′ viral genome that is capped and polyadenylated (early phase). Two polyprotein precursors (pp1a and pp1ab) are generated, the latter in fewer amounts and longer than pp1a because it is generated through −1 frameshifting that allows the stop codon terminating the synthesis of pp1a to be avoided. Programmed −1 translational frameshifting is conserved in all coronaviruses. It is necessary for the synthesis of the viral RNA-dependent RNA polymerase (RdRp/Nsp12) and the downstream viral non-structural proteins (Nsp) that encode the core enzymatic functions involved in the capping of viral RNA, RNA modification and processing, and RNA proofreading [56]. 

The importance of three-stemmed pseudoknot-dependent −1 ribosomal frameshifting has been suggested for the propagation of SARS-related coronaviruses. This process has never been observed in any endogenous human transcript in human cells and, therefore, could represent an opportune drug target with minimal tolerance for drug-resistant mutations [71]. Both these polyprotein precursors are immediately cleaved, generating 16 viral Nsps acting in this early phase. Proteolytic cleavage of pp1a and pp1ab is facilitated by the viral protease activity residing in Nsp3 (PLpro) and Nsp5 (3CLpro/Mpro). PLpro proteolytically releases Nsp1, Nsp2, Nsp3, and the amino terminus of Nsp4 from the polyproteins pp1a and pp1ab. Mpro proteolytically releases Nsp5–16 and the carboxy terminus of Nsp4 from the polyproteins pp1a and pp1ab [56]. The Nsp1 protein rapidly inhibits cellular protein synthesis [72] and indirectly induces cellular mRNA degradation, thus inhibiting the expression of newly synthesized cellular mRNA. In the meantime, viral RNA synthesis starts with the formation of the RNA transcription and replication complex (RTC) and double-stranded RNA (dsRNA) structures are produced early on during the infection cycle. Nsps also provide the biogenesis of viral replication organelles consisting of characteristic perinuclear double-membrane vesicles (DMVs), convoluted membranes (CMs), and small open double-membrane spherules (DMSs), creating a protective microenvironment for viral genomic RNA replication and transcription of subgenomic (sg) mRNAs. The characteristic nested set of coronavirus sg mRNAs allows the expression of the remaining 3′ one-third of the viral genome (late phase). At this later time, the ORFs encoding the structural proteins (spike (S), envelope (E), membrane (M), and nucleocapsid (N) in SARS-CoVs) are expressed together with the ORFs encoding for the so-called accessory proteins, interspersed between the structural ORFs. The accessory genes display a high variability among coronavirus groups and usually show no sequence similarity with other viral and cellular proteins. In addition, it has been observed some notable differences between the accessory proteins of SARS-CoV-2 (Orf3a, Orf3b, Orf3c, Orf3d, Orf6, Orf7a, Orf7b, Orf8, Orf9b, Orf9c, and Orf10: www.viralzone.expasy.org/8996 and www.viralzone.expasy.org/9076, accessed on January 2011) and SARS-CoV (ORF3a, ORF3b, ORF6, ORF7a, ORF7b, ORF8a, ORF8b, ORF9b, Orf14: www.viralzone.expasy.org/764, accessed on January 2011). ORF3d is an overlapping gene identified in SARS-CoV-2 [73]. In SARS-CoV-2, ORF3b contains a premature stop codon and is thus substantially shorter than the SARS-CoV variant studied. SARS-CoV-2 ORF8 shows a very low homology to SARS-CoV ORF8. The coding sequence of SARS-CoV ORF8 went through a gradual deletion over the course of the SARS-CoV epidemic. These few examples shed light on the complexity of coronavirus evolution. Overall, the ORF proteins have regulatory and immune evasion properties exerted in the late phase of the viral life cycle. In the meantime, translated structural proteins (S, M, and E) translocate into the endoplasmic reticulum (ER) membranes and transit through the ER-to-Golgi intermediate compartment (ERGIC), where the interaction with N-encapsidated, newly produced genomic RNA results in budding into the lumen of secretory vesicular compartments. Finally, virions are released from the infected cell by exocytosis [56].

## 4. SARS-CoV-2-Induced Cytokine Storm

Knowledge of SARS-CoV-2 replication, gene function, and host interactions is quickly accumulating. As for the host–pathogen interaction, rapid and uncontrolled viral replication of SARS-CoVs is linked to viral evasion of the host’s innate immune response throughout the viral cycle, starting from its initial steps. The aberrant pro-inflammatory responses and immune cell infiltration in the lungs provoke tissue damage and contribute to the clinical manifestation of COVID-19. Disease severity depends on viral infection and is deeply influenced by the host’s immune response. When SARS-CoV-2 infects target cells, the active replication and release of the virus cause pyroptosis and release PAMPs and DAMPs, including ATP and nucleic acids [74]. These are recognized by PRRs of neighboring epithelial cells, endothelial cells, and alveolar macrophages, triggering the generation of pro-inflammatory cytokines and chemokines. These proteins attract other immune cells, including T cells, to the site of infection, promoting further inflammation [75]. Usually, virus-specific T cells eliminate the infected cells before the virus spreads [76]. Neutralizing antibodies in these individuals can block viral infection, and alveolar macrophages recognize opsonized viruses and apoptotic cells and clear them by phagocytosis. Altogether, these processes lead to virus clearance and minimal lung damage, resulting in recovery. However, in some individuals, the recruitment of leucocytes to the infection site may lead to an overproduction of pro-inflammatory cytokines [77], which can damage the lung tissue [78]. Furthermore, the resulting cytokine storm circulates to other organs, leading to multi-organ damage [79,80,81]. Therefore, the immune response to SARS-CoV-2 unpredictably deviates towards inflammatory tissue damage, leading to rapid evolution from moderate to severe disease characterized by progressive pneumonitis, ARDS, and multiorgan failure with fatal outcomes.

Soluble and cellular mediators contribute to these two opposite outcomes, although the mechanisms that tilt the balance between the protective and deleterious immune response are not fully understood. One hypothesis is that in the absence of a robust and rapid antiviral response, which might be due to virus-triggered immune evasion strategies or pre-existing medical and/or genetic conditions, the ongoing infection promotes an exaggerated production of cytokines and chemokines, leading to the activation and the recruitment of different immune cell subsets with subsequent local or systemic organ damage [77]. Therefore, the highly pathogenic potential of SARS-CoV-2 can rely on the peculiar ability to hamper the IFN pathway and, on the other hand, to stimulate an elevated production of proinflammatory chemokines and cytokines, in particular IL6 [77]. This also implies that, in a different manner from SARS-CoV, SARS-CoV-2 replicates more actively and effectively in human lung tissues, where a higher viral load was found likely due to ongoing immune evasion mechanisms or defective viral clearance [82].

The elevated serum concentration of TNF-α, IL-6, and IL-10 detected in severe COVID-19 patients is associated with a reduced number of circulating T cells that, in addition, show an exhausted phenotype characterized by high expression of immune checkpoint molecules such as programmed cell death protein 1 (PD-1) and T-cell immunoglobulin and mucin domain containing-3 (Tim-3) [83]. An important feature of COVID-19 immunopathogenesis emerged from a longitudinal study conducted in patients with moderate and severe disease displaying a similar expression profiling of inflammatory cytokines up to 10 days after the disease onset, while, at later time points, TNF-α, IL-6, and IL-10 levels steadily declined in patients with moderate disease and instead remained elevated in those with severe COVID-19 [84].

Single-cell analysis conducted in bronchoalveolar lavage fluids (BALFs) from severe patients showed a higher percentage of macrophages and neutrophils but a smaller amount of mDC, pDC, T, and NK cells when compared to mild cases [85]. Xu et al. [86], using single-cell RNA sequencing, characterized the peripheral blood mononuclear cells (PBMCs) from uninfected controls and COVID-19 patients and cells in paired broncho-alveolar lavage fluid (BALF). They found a close association between decreased DCs and increased monocytes resembling myeloid-derived suppressor cells (MDSCs), which correlated with lymphopenia and inflammation in the blood of severe COVID-19 patients. A huge expansion of immune suppressive MDSC was confirmed by several groups, demonstrating an association between MDSC frequency and COVID-19 outcome (reviewed in [87]).

Monocyte-macrophages in BALFs of COVID-19 patients produced massive amounts of cytokines and chemokines. The frequencies of peripheral T cells and NK cells were also significantly decreased in severe COVID-19 patients, especially for innate-like T and various CD8+ T cell subsets, compared to healthy controls. In contrast, the proportions of various activated CD4+ T cell subsets, including Th1, Th2, and Th17-like cells, were increased and more clonally expanded in severe COVID-19 patients. The patient’s peripheral T cells showed no sign of exhaustion or augmented cell death, whereas T cells in BALFs produced higher levels of IFN-γ, TNF-α, CCL4, and CCL5. Paired TCR tracking indicated abundant recruitment of peripheral T cells to the severe patients’ lungs. Collectively, these data suggest that during severe COVID-19, lung monocyte-macrophages are prone to produce chemokines that recruit more monocytes and neutrophils, which, once migrated into the infected lung, contribute to the excessive production of proinflammatory cytokines [85].

As reviewed by Ricci et al. [88], the reduced peripheral NK cell counts and impaired cytotoxic activity observed in severe SARS-CoV-2-infected subjects with respect to mild cases and in deceased versus survivor patients, significantly parallel with the increase in IL-6 circulating levels. These observations suggest that functional impairment of NK activity might be due to an enhanced innate immune cell activation with massive proinflammatory cytokine production. In addition, blood samples from severe patients are characterized by a high neutrophil-to-lymphocyte ratio (NLR), a widely used marker of inflammation and infection. Interestingly, Combes et al. [89] showed that a neutrophil subpopulation expressing an ISG signature was strongly represented in patients with mild/moderate COVID-19 but not in patients with severe COVID-19 [89]. However, Neutrophil Extracellular Traps (NET) dysregulation was found in COVID-19 patients who showed elevated serum levels of cell-free DNA, myeloperoxidase DNA, and citrullinated histone H3, specific markers of NETs. Interestingly, in vitro experiments demonstrated that the exposition of neutrophils from healthy controls to COVID-19 patient sera promotes NET release [90]. Excessive NET formation can trigger inflammation and thrombosis, which results in permanent organ damage.

Despite the cytokine storm, no measurable IFN-β and low levels of IFN-α and ISGs were associated with a higher blood viral load and inflammatory response in sera of severe and critical COVID-19 patients compared to mild cases [91]. The elevated ISG expression in peripheral blood mononuclear cells (PBMC) of mild or asymptomatic versus severe COVID-19 patients likely depends on an early and robust IFN production in the lungs that subsequently diffuses into the bloodstream where high IFN-α plasma levels are found [92,93]. The early interaction between SARS-CoV-2 and immune cells was investigated by interrogating an in vitro human peripheral blood mononuclear cell (PBMC)-based experimental model [92]. Even without a productive viral replication, the virus mediates a vigorous TLR7/8-dependent production of type I and III IFNs and inflammatory cytokines and chemokines, known to contribute to the cytokine storm observed in COVID-19. This type I IFN was released by pDC via an ACE-2-independent, but Neuropilin-1-dependent, mechanism. Viral sensing regulates pDC phenotype by inducing cell surface expression of PD-L1 marker, a feature of type I IFN-producing cells. Coherently to what was observed in vitro, asymptomatic SARS-CoV-2 infected subjects displayed a similar pDC phenotype associated with a very high type I IFN serum level and induction of antiviral IFN-stimulated genes in PBMC. Conversely, hospitalized patients with severe COVID-19 display a shallow frequency of circulating pDC with an inflammatory phenotype and high serum levels of chemokines and pro-inflammatory cytokines [92,93,94].

Vanderheiden et al. [5] used primary human airway epithelial cells isolated from the bronchial or tracheal region to evaluate the response of primary airway cells to the virus. They are highly susceptible to SARS-CoV-2, and similarly to SARS-CoV, virus replication occurs primarily in ciliated cells, where ACE-2 expression is localized. Compared to mock-infected cells, SARS-CoV-2-infected cells have significant enrichment for TNF-alpha and IL-6-STAT-3-related signaling, indicating that in vitro SARS-CoV-2 infection induces a proinflammatory phenotype in these cells as happens in vivo. NF-kB and ATF-4 were consistently identified as top predicted transcriptional regulators following SARS-CoV-2 infection. Accordingly, SARS-CoV-2-infected cells had increased transcript expression of IL-6, TNF-α, and other cytokine genes, including the IL-17 family (IL17C and IL-23A) and the IL-1 family (IL-18 and IL1-β). In addition, several chemokines were upregulated, including molecules that promote the migration of monocytes (CCL4 and CCL5) and neutrophils (CXCL8 and CXCL6). SARS-CoV-2 infection does not induce a type I or III IFN response. However, cell pre-treatment with type I or III IFN increases antiviral activity and restricts SARS-CoV-2 replication, indicating that the virus is susceptible to type I and III IFNs if the antiviral state is induced before the infection. RNA-Seq data also suggest that SARS-CoV-2 may induce an immune response ineffective against viruses, as evidenced by a preference to produce cytokines whose primary function is the defense against extracellular pathogens and the complete lack of type I and III IFN signaling [5]. Taken together, these results indicate a maladapted immune response profile associated with severe COVID-19 and poor clinical outcomes. SARS-CoV-2 can hit the main early hub of the host’s innate immune response and break up a coordinated and effective immunity required to resolve the infection [95].

## 5. Genetic Background of the IFN System and SARS-CoV-2 Susceptibility

Many efforts have been established to elucidate the role of host genetic factors in SARS-CoV-2 susceptibility and COVID-19 severity (the COVID Human Genetic Effort [96] and the COVID-19 Host Genetics Initiative [97]). Zhang et al. [98] tested the hypothesis that monogenic inborn errors in three loci identified as mutated in patients with life-threatening influenza (TLR3, IRF7, and IRF9) and 10 loci mutated in patients with other viral illnesses could also underlie life-threatening COVID-19 pneumonia. Genetic screening of 659 patients with severe COVID-19 and 534 individuals with moderate or asymptomatic infection revealed an enrichment in defective functional variants in 13 loci in the first group of patients. In 23 patients (3.5%), autosomal recessive (AR) deficiencies (IRF7 and IFNAR1) and autosomal dominant (AD) deficiencies (TLR3, UNC93B1, TICAM1, TBK1, IRF3, IRF7, IFNAR1, and IFNAR2) were identified. In parallel, Bastard et al. [99] tested the hypothesis that anti-type I IFNs antibodies (Abs) may underlie severe COVID-19. The authors found that 135 of 987 patients (13.7%) with life-threatening COVID-19 pneumonia had IgG anti-IFN-ω, IFN-α, or both. These IgG had the ability to neutralize IFNs both in vivo and in vitro in 101 patients (10.2%) and were absent in individuals with asymptomatic or mild infection. Interestingly, anti-IFN Abs were present in 4 of 1227 (0.33%) healthy individuals. Indeed, variants with the most significant impact on COVID-19 outcomes are expected to be rare in the population. Studying rare variants may provide additional insights into disease susceptibility and pathogenesis, thereby informing therapeutics development. 

Recently, Butler-Laporte et al. [100] combined whole-exome and whole-genome sequencing from 21 cohorts across 12 countries and performed rare variant exome-wide burden analyses for COVID-19 outcomes. Analyzing 5085 severe disease cases and 571,737 controls, they observed that the presence of a rare deleterious variant in the SARS-CoV-2 sensor toll-like receptor TLR7 (on chromosome X) was associated with a 5.3-fold increase in developing severe disease (95% CI: 2.75–10.05, *p* = 5.41 × 10^−7^), confirming the importance of TLR7 to recognize SARS-CoV-2 infection. Lee et al. [101] reported autosomal recessive deficiencies of OAS1, OAS2, or RNASEL, well known ISG, in five unrelated children with the multisystem inflammatory syndrome (MIS-C), a rare and severe condition that follows benign COVID-19 in children. The cytosolic dsRNA-sensing OAS1 and OAS2 generate 2′-5′-linked oligoadenylates (2-5A) that activate the ssRNA-degrading RNase L. Monocytic cell lines and primary myeloid cells with OAS1, OAS2, or RNASEL deficiencies produce excessive amounts of inflammatory cytokines upon dsRNA or SARS-CoV-2 stimulation. Exogenous 2-5A suppresses cytokine production in OAS1- but not RNase L-deficient cells. Cytokine production in RNase L-deficient cells is impaired by MDA5 or RIG-I deficiency and abolished by MAVS deficiency. Recessive OAS-RNase L deficiencies in these patients unleash the production of SARS-CoV-2-triggered, MAVS-mediated inflammatory cytokines by mononuclear phagocytes, thereby underlying MIS-C.

This genetic evidence demonstrates the importance of type I and III IFN systems and of ISGs in triggering the natural resolution of the infection and supports the idea that counteraction of the IFN system by the virus represents an essential mechanism to subvert the correct development of a protective immune response.

## 6. SARS-CoV-2 Evasion of the IFN System

The ability of SARS-CoV-2 variants of concern (VOC) to evade IFN-mediated immune response is highlighted by different inhibitory effects elicited by 17 human IFNs tested in vitro against different viral lineages, including ancestral and five major VOC that include the B.1.1.7 (alpha), B.1.351 (beta), P.1 (gamma), B.1.617.2 (delta), and B.1.1.529 (omicron) lineages. Compared to ancestral isolates, SARS-CoV-2 VOCs exhibited increased IFN resistance, further suggesting that evasion of innate immunity could be significant and is an ongoing driving force for SARS-CoV-2 evolution. The increased virus fitness associated with VOCs is the result of a complex interplay between virus biology and human immunity changes due to vaccination and prior infection that also influence the immune evasion of the IFN system [102]. These findings have implications for the increased transmissibility and/or lethality of emerging variants [103].

Type I and type III IFNs represent the first line of immune defense against viral mucosal infections. Several, if not all, SARS-CoV-2 proteins demonstrate at least a mild inhibitory activity on type I and III IFN production and/or responses also affecting type II, i.e., IFN-ϒ response. It has been demonstrated that SARS-CoV-2 contrasts type I and III IFN induction, type I IFN receptor, STAT-1 and 2-mediated JAK-STAT signal transduction, and function of some ISG during both the early and late phase of the viral cycle. Type I IFN, induced by the activation of the cGAS/STING pathways via the release of mitochondrial DNA due to mitochondrial damage, appears to have deleterious instead of protective effects [104]. STING is activated by SARS-CoV-2 infection in damaged lung epithelia and macrophages, and its activation is associated with increased pathology. Although rapid and early induction of type I IFNs limits virus propagation, a sustained increase in its levels in the late phase of the infection is associated with aberrant inflammation and poor clinical outcome [104]. These data confirm complex interplay among SARS-CoV-2, IFNs, and the overall immune response.

**Figure 4:**
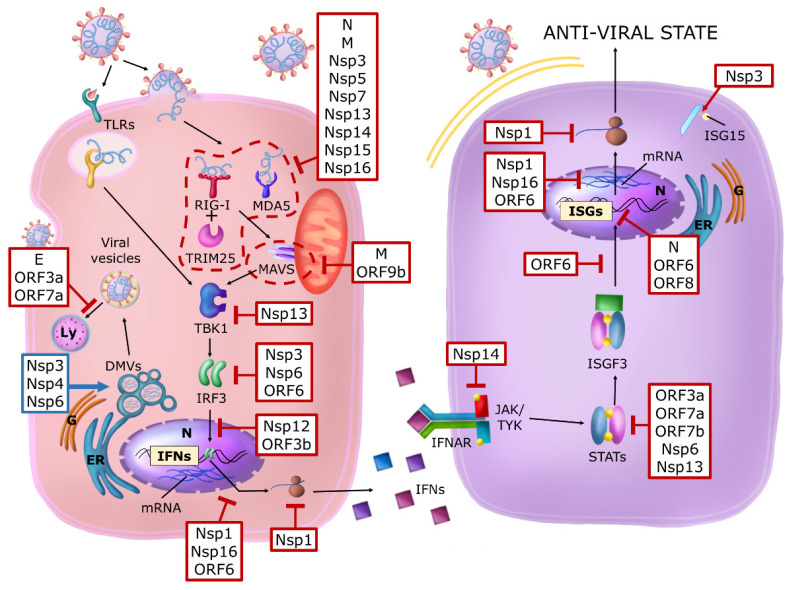
SARS-CoV-2 evasion of the IFN system. Schematic representation of viral proteins interfering with the signaling pathways leading to Type I and III IFNs production (**left**) and IFN responses (**right**). (See Section 6 for details).

To define which and how viral products impact the IFN system, many classical approaches were employed based on: (1) transfection of different cell lines with plasmids expressing reporter genes driven by IFN-β or ISG promoters together with expression vectors for non-structural, structural and accessory viral proteins or also infected with RNA viruses; (2) expressing cellular proteins, such as RIG-I, MAVS, TBK-1/IKK-ε, and constitutively active IRF-3, able to induce IFN-β transcription at different key points in the signaling pathway, or stimulated with IFN-β. However, overproducing a single viral protein might not trigger the same cellular effects as a natural infection by the virus. Verifying the findings requires experiments with the virus itself, genetically tweaked to lack individual proteins that require elaborate biosafety precautions. In-depth reviews on this topic have been published during the pandemic considering new results that are continually being achieved, refining our knowledge (i.e., refs. [88,95,101,105,106,107,108,109,110]. Figure 4 summarizes the main targets of the principal viral proteins that interfere with IFN pathways. Below, we provide an updated overview of the different results.

Many viral proteins, named Nsp1-16, are produced immediately after virus entry. They are generated via the rapid proteolytic maturation of the pp1a and pp1ab polyprotein precursors generated by the genome translation. These proteins are involved in starting transcription and replication of the viral genome and in the first interaction with the cell machinery. In the meantime, type I and III IFN induction might be induced via PRR that, sensing the virus, might activate NF-κB and IRF3 transcription factors through TRAF3, TBK1, and the IKKε. NF-κB participates in IFN-β and -λ genes transcription, but also in the production of pro-inflammatory cytokines (e.g., IL-1, IL-6, and TNF-α). Upon viral infection, type III IFN is the most abundant in the mucosal, respiratory, gastrointestinal, and urogenital tracts (reviewed in [108]). They are secreted by epithelial cells, macrophages, CD8+ T lymphocytes, NK cells, Treg lymphocytes, dendritic cells, and hepatocytes [111]. At least during the respiratory infection caused by the type A influenza virus, it seems to be produced earlier [112]. 

Regarding SARS-CoV-2, upon infection of intestinal epithelial cells, using both colon-derived cell lines and primary colon organoids, type III IFN response appears more efficient than type I at controlling viral replication [113,114]. The PRRs contributed to the induction of type III IFN expression overlapping with those eliciting type I IFN expression. However, the selective type III IFN expression, not type I IFN, is also mediated by Ku70 (a cytosolic DNA sensor) [115]. Differences between type I and type III IFN in the gene promoter support the higher dependence of type III IFN on NF-κB that is instead not required for IFN-αs expression but necessary for IFN-β [116]. Likewise, type III IFN production involves MAVS signaling from the peroxisome, highly abundant in epithelial cells, in contrast to the mitochondrial location for IFN-β expression [8].

How does the early phase viral protein contrast the activation and action of IFN-mediated innate immune response? Nsp1 is a potent virulence factor that inhibits cellular protein synthesis, reducing ribosome pools that engage cellular mRNAs. Cryo-EM structure of the Nsp1-40S ribosome complex showed that Nsp1 inhibits translation by plugging mRNA entry into the channel of the 40S [72,117], therefore inhibiting the translation of IFNs and ISG mRNAs but allowing viral protein synthesis. In addition, the Nsp1 protein of SARS-CoV-2 interacts with the host mRNA export receptor heterodimer NXF1-NXT1, which is responsible for the nuclear export of cellular mRNAs [118]. Nsp1 prevents proper binding of NXF1 to mRNA export adaptors and NXF1 docking at the nuclear pore complex. As a result, a significant number of cellular mRNAs are retained in the nucleus during infection, including those for IFN and ISG production induced via virus sensing [119].

Nsp3 (PLpro), the papain-like protease involved together with Nsp5 (3CLpro/Mpro), the 3-chymotrypsin-like protease, in the fundamental pp1a and pp1ab polyproteins processing, has been reported to have de-ISGylation activity [120]. ISG15 is a ubiquitin-like modifier that regulates many cellular pathways and is induced by type I and III IFN [121,122]. It is a 17 kDa precursor protein rapidly processed into its mature 15 kDa form via protease cleavage to expose a carboxy-terminal motif, which allows the covalent binding of ISG15 to target proteins by the three-step process referred to as ISGylation. ISG15 is also an unconjugated protein that mainly localizes in the cytoplasmic fraction. It can be released into the extracellular milieu via non-conventional secretion (it lacks a secretory signal peptide). ISG15 has been found in neutrophil granules, microvesicles, and exosomes originating from TLR3-activated human brain microvascular endothelial cells or released via apoptosis.

Regarding its activity as an antiviral restriction factor, ISG15 inhibits the HIV budding process ISGylating TSG101, a component of the ESCRT-I complex, thus inhibiting its ability to target Gag to favor the budding viral process from the cellular membranes. The host cell ISGylates the NS1 protein of the avian influenza A virus, impeding its interactions and limiting its immune-evasion actions. Human patients with recessive inheritance ISG15 deficiency (ISG15−/−) do not appear to be more susceptible to viral infection. However, the lack of mycobacterium-induced ISG15 secretion by leukocytes-granulocyte reduced the production of IFN-γ by lymphocytes, including natural killer cells, enhancing susceptibility to mycobacterial disease [123]. In addition, ISGylation of the caspase activation and recruitment domains (CARD) of the cytoplasmic dsRNA sensor MDA5 promotes its oligomerization, thereby triggering activation of innate immunity against a range of viruses, including coronaviruses, flaviviruses, and picornaviruses [124,125,126].

SARS-CoV-2 antagonizes the ISG15-dependent activation of MDA5 through direct de-ISGylation mediated by the protease Nsp3 (PLpro) [122]. IRF3 is also a target of ISGylation that induce its stabilization, prolonging the IFN response [127]. Moreover, Nsp3 (PLpro) can cleave IRF3 contributing to the blunted type-I IFN response seen during SARS-CoV-2 infections. Nsp5(3CLp)-mediated cleavage of NLRP12 and TAB1 points to a molecular mechanism for enhanced production of cytokines and the inflammatory response observed in COVID-19 patients [128]. In contrast to ubiquitin (Ub), ISG15 requires IFN production to be efficiently conjugated to other proteins because the ISG15 conjugating enzymes are ISG themselves. Moreover, despite the multitude of E3 ligases for Ub-modified targets, a single E3 ligase termed HERC5 (in humans) is responsible for the bulk of ISG15 conjugation. After controlling the pathogen assault, cells must decelerate the ISGylation pathway to avoid the risk of chronic inflammation or even cell death. For this purpose, cells encode an endogenous de-ISGylation enzyme, USP18, that removes ISG15 from conjugates while releasing unconjugated ISG15. Specifically, SARS-CoV, MERS-CoV, and SARS-CoV-2 encode Nsp3 (PLpro) that bear striking structural and catalytic similarities to the catalytic core domain of eukaryotic deubiquitinating enzymes of the Ubiquitin-Specific Protease (USP) sub-family. The cleavage specificity of these PLpro enzymes is for flexible polypeptides containing a consensus sequence (R/K)LXGG, enabling them to function on two seemingly unrelated categories of substrates: (1) the viral polyprotein 1 (PP1a, PP1ab) and (2) Ub- or ISG15-conjugates. A narrow cleft restricts access to the active site of PLpro to glycine at positions P1 and P2, limiting substrates to specific sequences found in the viral polypeptide precursors (PP1a, PP1ab) or in the flexible C-termini of Ub and ISG15. Consequently, PLpro enzymes from MERS, SARS, and SARS-CoV-2 can hydrolyze the isopeptide bond at either the C-terminus of Ub or ISG15. Even if MERS-CoV, SARS-CoV, and SARS-CoV-2 PLpros can all cleave conjugated Ub or ISG15, SARS-CoV-2 PLpro prefers ISG15 as a substrate [120,129]. De-conjugating ISG15, the virus also creates free ISG15, which may affect the immune response in two opposite pathways: free ISG15 negatively regulates IFN signaling in humans by binding non-catalytically to USP18, and at the same time, free ISG15 can be secreted from the cell. Extracellular ISG-15 binds to LFA-1 on NK cells inducing IFN-gamma production [130]. Therefore, small-molecule inhibitors of PLpro might have a dual therapeutic effect of inhibiting the cleavage of viral polyproteins and blocking intracellular de-ISGylation. 

Furthermore, inhibiting PLpro would also limit the secretion and extracellular signaling of ISG15. Interestingly, it has been reported that the non-covalent inhibitor GRL-0617 that inhibits the catalytic activity of SARS-CoV-2-PLpro could restore ISGylation of host proteins and was sufficient for SARS-CoV-2 infected cells to recover their IFN-signaling and decrease the number of viral particles observed in the supernatant [129]. Limiting ISG15 secretion might be expected to be detrimental in combating the infection because pro-inflammatory cytokines are important in recruiting and activating cells of the adaptive immune response.

On the other hand, if some of the most severe and deadly consequences of infection are a result of cytokine release syndrome, then limiting ISG15 secretion and signaling by therapeutically inhibiting PLpro might be beneficial [131]. PLpro also mediates DAXX re-localization to cytoplasmic sites and promotes its proteasomal degradation [132]. DAXX is a scaffold protein residing in PML nuclear bodies and is able to limit SARS-CoV-2 replication [34].

Recently, Song et al. [133] described that Nsp5 (3CLpro/Mpro) inhibits ISG induction cleaving histone deacetylases, thus influencing cellular transcription, and abolishes the activity of the ISG DCP1A (decapping mRNA 1A) cleaving it at residue G343.

Other Nsps avoid recognition of vRNA by dsRNA sensors RIG-I and MDA5 (reviewed in [95]). Nsp3, 4, and 6 drive the formation of DMV shielding replicating vRNA from RLR recognition [134].

Nsp 7 inhibits type I and III production targeting RIG-I/MDA5, TRIF, and STING signaling pathways [135]. Moreover, capping of the viral RNA carried out by Nsp13, 14, and 16 also circumvents recognition by both RIG-I and MDA5. MDA5 sensing can be further evaded by shortening and preventing the accumulation of 5′-polyU-containing negative-sense viral RNA through the action of Nsp15 and by Nsp5 (3CLpro/Mpro)-mediated inhibition of the TRIM25-RIG-I interaction.

In addition, Nsp16, a ribonucleoside 2′-O-methyltransferase catalyzing the transfer of a methyl group to mRNA as part of the capping process of the viral mRNA, evades IFIT1 and IFIT3 restriction. IFIT1 and IFIT3 are IFN-stimulated genes that sense the lack of 2′-O-methylation [136].

Many adaptors in the type I IFN pathway and inflammatory response are regulated by autophagic cargo receptor p62, such as the stimulator of IFN genes (STING) and are absent in melanoma 2 (AIM2). Nsp13 inhibits type I IFN production by recruiting TBK1, the adaptor kinase important in the induction of type I IFN and proinflammatory cytokines, to p62 for autophagic degradation, enabling it to evade the host’s innate immune response. TBK1 and SARS-CoV-2 Nsp13 form a p62-associated complex that enters autophagosome to degrade TBK1 after fusing with lysosome [137]. SARS-CoV-2 early-phase proteins can also inhibit IFNs action [95]. Nsp14, which has replication proof-reading activity and controls the viral RNA capping, inhibits the expression of IFNAR1, thus impairing STAT-1 tyrosine phosphorylation induced by IFN-β. Nsp6 blocks IRF3 activation and inhibits STAT-1 and -2 tyrosine phosphorylation, whereas Nsp13 blocks STAT-1 and -2 tyrosine phosphorylation. Nsp12, the RNAdRNAp, inhibits IRF3 nuclear translocation. SARS-CoV-2 Nsp15 inhibits de novo autophagy induction but is less efficient in blocking type I IFN induction and signaling compared to SARS-CoV1 Nsp15. Nsp16 localizes into the nucleus and binds snRNA U1 and U2 components of the pre-mRNA spliceosome, increasing the level of ISG RNAs exhibiting intron retention [138].

Soon after the appearance of the subgenomic mRNA, four structural viral proteins and new non-structural proteins, named ORFs, are generated. In this later phase of the viral life cycle, viral proteins are involved in assembling and releasing the viral particles and maintaining a robust immune evasion. Again, the IFN system is one important target. The N nucleocapsid structural protein that will wrap the viral genome binds the DExD/H domain of RIG-I, inhibiting the activation of IRF3 and IFN-β promoter. The membrane tetraspanning protein M, inserted in the envelope of the viral particles, was reported to antagonize RLR signaling, abrogating the induction of IFN-β and -λ expression. In vitro M interacts with RIG-I, MDA5, MAVS, TBK1, and TRAF3, but not IRF3. By interfering with the prion-like aggregation of MAVS and its association with SNX8, it seems to disrupt the correct recruitment of the downstream components TRAF3, TBK-1, and IRF3 to the MAVS complex. This phenomenon has also been described in other infections. In vitro infection with Sendai Virus (SeV) showed that M co-localizes with TBK1 and inhibits IRF3 phosphorylation and nuclear translocation [106,139,140]. TBK1 is a pivotal crossroad for multiple signaling pathways beyond type I and III IFN induction, regulating proinflammatory signal and autophagy [141], thereby representing an essential target of SARS-CoV-2 proteins.

Post-translational modifications, such as glycosylation, phosphorylation, and acylation, related to SARS-CoV-2 proteins are conservative and pathogenic [142]. Acetylated envelope E protein of SARS-CoV-2 interacts with BRD2/4 (bromodomain-containing protein 2/4) to influence the host immune response. BRD2/4 is a member of the BET domain family of epigenetic readers. Acetylated E disrupting the BRD-acetylated histone interaction modulates the protein expression beneficial to the virus. The C-terminal region of the SARS-CoV-2 E protein mimics the N-terminal segment of histone H3 and is an interacting partner of bromodomains [143]. E, ORF3a, and ORF7a block autophagic turnover: ORF3a and ORF7a exerted the most potent autophagy antagonism, targeting the late endosome pathway by blocking the fusion of lysosomes with autophagosomes and by decreasing lysosomes acidification respectively. ORF3a inhibits cGAS-STING-mediated autophagy flux and antiviral function [144]. In addition, ORF3a, ORF7a, and ORF7b disrupt the phosphorylation of STAT-1 and STAT-2 after 30 min of IFN-α treatment. ORF7a and ORF7b preferentially inhibit STAT-2 phosphorylation with respect to STAT-1 (reviewed in [109]).

SARS-CoV-2 ORF7a has also been described to counteract the antiviral effect of Serine Incorporator 5 (SERINC5) by blocking the incorporation of over-expressed SERINC5 in budding virions [145]. SERINC5 is a cellular multi-pass transmembrane protein involved in sphingolipid and phosphatidylserine biogenesis and potently restricts several retroviruses, including Human Immunodeficiency Virus (HIV). SERINC5 is incorporated in the budding virions leading to the inhibition of virus infectivity. Proteins of other viruses, in particular the Nef protein of HIV, S2 of equine infectious anemia virus (EIAV), and the GlicoGag protein of ecotropic murine retroviruses counteract the antiviral effect of this restriction factor [146,147,148,149]. ORF3b of SARS-CoV-2 is markedly shortened compared with the one of SARS-CoV. The lack of a putative nuclear localization signal strongly antagonizes the IFN responses and impairs IRF3 nuclear translocation [150]. ORF9b disrupts RLR (Rig-I-like Receptors) signalosome by localizing in the mitochondria, thus preventing TBK-1 activation, IRF3 phosphorylation, and nuclear translocation. In addition, it associates with the antiviral modulator NEMO and interrupts K63-linked polyubiquitination, thereby inhibiting NF-κB signaling and suppressing IFN-β induction and pro-inflammatory cytokines expression. 

ORF9b associates with components of the cGAS-STING DNA sensing pathway, decreasing TBK1 and IRF3 activation [151,152,153] (reviewed in [95] and in [109]). ORF6, ORF8, and N proteins are also inhibitors of the type I interferon pathway. Indeed, these three proteins showed strong inhibition of IFN-β and NF-κB- and ISRE-responsive promoters. Notably, the potent antagonism of ORF6 towards the IFN response is a conserved function across SARS-CoV viruses. Comparative experiments with SARS-CoV-2 and SARS-CoV identified ORF6 as the most consistent inhibitor of the type I IFN system among the two related coronaviruses [154]. The ORF6 protein of SARS-CoV-2 presented a substantial similarity with the ORF6 protein of SARS-CoV-1 and bat-CoV-SL-RmYN02 (68.9 and 70.5%, respectively), but even more with the ORF6 protein of the bat-SL-CoV-RaTG13 and Pangolin-CoV-2019 (100 and 96.7%) [155]. ORF6 localizes to the nuclear pore complex (NPC) and exerts its function by binding to Nup98-Rae1; this association impairs importin karyopherin alpha (KPNA)2-mediated nuclear translocation of activated IRF3 and ISGF3/STAT-1 [107,156,157].

ORF6, interacting with cellular Rae1, also inhibits cellular protein production by blocking mRNA export, including those encoding antiviral factors such as IRF1 and RIG-I [158]. Altogether, these results indicate that ORF6 can disrupt host cell innate immune signaling inhibiting the earliest stages of innate signaling by downregulating the expression of both pathogen recognition receptors (PRRs) and antiviral transcription factors, such as IRF1.

To understand the innate cellular control of viral infection and their potential impact on COVID-19 outcomes, Martin-Sancho et al. [159] conducted a comprehensive evaluation of ISGs able to inhibit SARS-CoV-2 infection. They conducted a gain-of-function screen using 399 human ISGs induced in human tracheobronchial epithelial and human alveolar epithelial A549 cells by universal type I IFN and ISG described to have wide antiviral activity. The 399 ISGs’ capability to inhibit SARS-CoV-2 replication was evaluated using ectopic expression screening in the human cell line 293T. 293T cells can be efficiently transfected, support productive replication of SARS-CoV-2 when expressing the viral entry factors ACE2 and TMPRSS2 and respond to IFN treatment. 293T cells were transfected with individual ISGs along with ACE2 and TMPRSS2 for 30 h and then challenged with SARS-CoV-2 at a low multiplicity of infection. The results revealed that restriction of SARS-CoV-2 is mediated by a limited subset of 65 ISGs, mostly endoplasmic reticulum (ER)- and Golgi-resident proteins known as regulators of ER-associated protein degradation (ERAD), lipid membrane composition, and vesicle transport. Among the inhibitory ISGs, they also identified BST2/Tetherin, which inhibits the viral egress of enveloped viruses and is antagonized by the SARS-CoV-2 accessory protein Orf7a. Finally, post-transcriptional modification of the viral proteins influences their ability to counteract host response [142].

All the data emphasize the importance of type I and III IFN in contrasting SARS-CoV-2 infection and indicate that avoidance of the IFN system is a critical factor in the virus evolution. Indeed, the high number of viral proteins involved in counteracting viral sensing by PRR and IFN production and action ensures that the swarm of mutants produced during viral replication cannot lose the ability to balance this important innate response pathway.

## 7. Antiviral Treatments against COVID-19

The outcome of infection with the SARS-CoV-2 varies a lot, from asymptomatic to severe disease and death. Severe disease is characterized by poor oxygen saturation and massive inflammatory responses in the lung, leading to severe pneumonia and ARDS, and, in the most serious cases, multiorgan failure [2]. Therefore, great effort has been contributed to the development of treatments aimed at counteracting severe COVID-19.

As the first specific defense against SARS-CoV-2 infection, different vaccines have been developed to induce the production of neutralizing antibodies. Up until now, none of them was able to induce sterilizing immunity, even if they reduce pathogenicity. In the meantime, pharmacological approaches have emerged due to the intensive search for antiviral molecules that can inhibit the action of specific viral proteins inhibiting viral entrance, replication, and/or assembly [160,161]. The approaches could become more efficient by combining molecules with different mechanisms of action to prevent the appearance of resistant variants due to the high frequency of mutation that characterizes RNA viruses.

Drug developments to address the COVID-19 pandemic used four different strategies: (1) blocking viral structural proteins, thus inhibiting virus–host interactions, viral entry, and/or capsid assembly; (2) inhibiting the enzymes involved in the synthesis and replication of viral RNA and the polyprotein precursor maturation; (3) targeting viral virulence factors that mediate the host’s immune system escape; and (4) targeting direct interactions between viral and host proteins.

Most clinical trials were aimed at repurposing existing drugs against COVID-19. Broad-spectrum antivirals such as Lopinavir/Ritonavir and Remdesivir have been used in clinical trials to check the efficacy against COVID-19. Lopinavir and Ritonavir (antivirals used against HIV), two protease inhibitors, have shown promising inhibitory activity against SARS-CoV-2. However, multiple controlled trials showed that the clinical efficacy of lopinavir/ritonavir on COVID-19 was poor and caused serious gastrointestinal adverse reactions [162].

A more effective inhibitor was subsequently identified; this molecule, nirmatrelvir [163,164,165], significantly reduced hospitalization and death. Nirmatrelvir inhibits the SARS-CoV-2 protease Nsp5 (3CLpro/Mpro) that participate, together with the viral protease Nsp3 (PLpro), in the maturation of the polyprotein precursors pp1a and pp1ab, thus inhibiting the replication of the viral genome and the generation of mature early phase viral proteins. Nirmatrelvir is administered together with ritonavir (Paxlovid), which is an inhibitor of cytochrome P450 3A and CYP2D6, to boost and maintain plasma concentrations of nirmatrelvir [166,167]. Part of nirmatrelvir efficacy might be due to some restoration of IFN sensitivity. This finding suggests that a co-treatment with type I or III IFN types and the protease inhibitor might increase the antiviral activity. Indeed, a nice study reported in vitro antiviral synergism against SARS-CoV-2 variants of the co-treatment with IFN-β (Betaferon, Bayer) and EIDD-1931 (the active metabolite of molnupiravir that induces lethal mutagenesis during virus replication) or nirmatrelvir, but not with Remdesivir, a viral RNAdRNAp inhibitor [168]. These results open the possibility of a combination therapy IFN-β + SARS-CoV-2 Nsp5 (3CLpro/Mpro) inhibitor to increase treatment efficacy.

The administration of oral antiviral agents is more feasible early in infection. Such therapies, if given promptly, could help mitigate hospitalization burden, facilitate postexposure prophylaxis, and potentially minimize household transmission. Even though developing resistance to protease inhibitors is possible [169]. Moreover, with the emergence of novel variants, the rapid evaluation of resistance to antiviral therapies and vaccines is highly required. Remdesivir is one of the first antivirals repurposed to treat COVID-19 patients. It is an inhibitor of viral RdRp. It was found to be an effective antiviral against previous significantly pathogenic coronaviruses like SARS-CoV-1 and MERS-CoV [170]. The compound is an adenine nucleoside analog prodrug, which possesses appreciable affinity to the SARS-CoV-2 replicase/transcriptase complex, making it a potential antiviral for COVID-19 [171,172]. Remdesivir is recommended for COVID-19 treatment but needs to be administered intravenously, which limits its widespread use during the pandemic. Therefore, several oral analogs of remdesivir have been developed to address this issue, including GS-621763, ATV006, and VV116, and the activity of VV116 was recently compared with nirmatrelvir–ritonavir for oral treatment [173]. In this study, a total of 822 participants underwent randomization, and 771 received VV116 (384 participants) or nirmatrelvir–ritonavir (387 participants). The early administration of oral VV116 was comparable with nirmatrelvir–ritonavir in terms of shortening the time to sustained clinical recovery in participants with mild-to-moderate COVID-19 who were at elevated risk for progression to severe disease. A phase-2 open-label randomized controlled trial has been recently published regarding combination therapy of Interferon Beta-1b and Remdesivir, indicating that early treatment with IFN beta-1b and remdesivir was safe and better than remdesivir only in alleviating symptoms and in shortening viral shedding and hospitalization with earlier seropositivity in high-risk COVID-19 patients [174].

## 8. Type I and III IFN Treatment Options

The sensitivity of the virus to IFN treatment in cell culture and multiple viral strategies to inhibit IFN production inside the infected cells suggested the clinical use of type I and III IFN for COVID-19 treatment. In COVID-19 patients, decreased production of type I IFNs, and large amounts of pro-inflammatory mediators, primarily TNFα, ILs (e.g., IL-1β, IL-2, IL-6, IL-7, IL-10, IL -12, IL-18, IL-33) and chemokines can be observed. Factors influencing type I IFN production and sensitivity, such as sex, age, genetic defects in IFN signaling, or autoantibodies against type I IFNs, predispose some people to a more severe disease outcome, highlighting the protective role of these antiviral cytokines in the early phase of infection, as previously described. Although rapid induction of type I IFNs limits virus propagation, a sustained increase in the levels of type I IFNs in the late phase of the infection is associated with aberrant inflammation and poor clinical outcome [175]. Activation of the cGAS–STING pathway by DNA from damaged tissue induces type I IFN production at the later stages of infection, sustaining deleterious inflammation [104]. Several clinical trials have been conducted to examine the potential use of different type I IFN subtypes (i.e., α or β) and routes of administration for improving the clinical outcome of patients infected with the new coronavirus (reviewed by [109]). These results underlie the specific induction of beneficial (early) versus detrimental (delayed) type I IFN responses. Previous results with SARS-CoV or Middle East respiratory syndrome coronavirus (MERS-CoV) showed that IFN therapy is only effective when it is used as prophylaxis or in the initial phase of the infection, whereas at later stages, type I IFNs can be ineffective or even detrimental to the host [176,177]. To reach an adequate concentration in the upper and lower respiratory tracts and limit systemic exposure to type I IFN, localized routes of administration, such as nasal drops or nebulization, were also evaluated. 

Nebulized IFN-α2b, with or without Umifenovir (Arbidol), was tested on 77 COVID-19 patients. In this exploratory study, Zhou Q et al. [178] reported a significant reduction in the duration of detectable SARS-CoV-2 RNA and a shortening of high plasmatic IL-6 and C-reactive protein. Results from a phase II trial using nebulized IFN-β1a (SNG001) showed an odds reduction of developing severe disease in SNG001-treated patients than in the placebo groups [179]. Nevertheless, to avoid the exaggeration of inflammation, type I IFN administration is not recommended in the third stage of the disease [177,180,181,182]. Lu et al. [183] have recently summarized the current utilization of IFN-α. Overall, type I IFN therapies might be indicated at the early stage of infection to avoid uncontrolled viral replication and the cytopathic effects of the viruses; however, due to their strong ability to activate immune cells, exogenous type I IFNs are no longer recommended for the treatment of infections associated with extensive inflammation. The importance of administration timing has been highlighted by a report in which delayed IFN-β administration in MERS-CoV-infected mice exacerbated a pro-inflammatory state. It increased the infiltration of activated monocytes, macrophages, and neutrophils in the lung, ultimately resulting in a worse outcome (e.g., fatal pneumonia) compared to mice treated within one day after infection [184]. Thus, the IFN response timing relative to the virus replication seems to be a critical factor that may profoundly affect the disease course.

Park and Iwasaki [177] discussed the possible advantage of using the type III IFNs, as both a preventive and a therapeutic measure for COVID-19. As mentioned, type III IFN sensitivity is restricted to epithelial cells, hepatocytes, and some immune cells inducing an ISG signature like type I IFN. In addition, type I IFN signaling leads to a more rapid induction and decline of ISG expression with respect to type III IFN [9]; moreover, type III IFN appears to be less inflammatory in vivo [15,185]. These properties could induce a potent antiviral effect against SARS-CoV-2 in vivo with reduced side effects. A mouse model of influenza virus infection showed that the restricted IFN-λ receptor distribution and a lack of IRF1 induction result in influenza viral load decline without inflammatory side effects in mice treated with IFN-λ. By contrast, mice treated with type I IFN show impaired survival [186]. In phase II placebo-controlled randomized trial, pegylated IFN lambda (peg-IFN-λ) administered subcutaneously accelerated viral decline in outpatients with COVID-19, increasing the proportion of patients with viral clearance by day 7, particularly in those with high baseline viral load [187]. Peg-IFN-λ has the potential to prevent clinical deterioration and shorten the duration of viral shedding. Further, analyzing the effect of IFN-λ, Santer et al. [188] described that a single dose of peg-IFN-λ accelerates SARS-CoV-2 clearance without affecting virus-specific T-cell responses or antibody production in mild-to-moderate acute SARS-CoV-2 infection. Accordingly, Reis et al. [189] reported that the incidence of hospitalization or an emergency department visit was significantly lower among those who received a single dose of peg-IFN-λ than among those who received a placebo.

Compared to current antiviral treatments for COVID-19, peg-IFN-λ treatment appears broad-acting and effective with a single dose supporting future studies as an early treatment option.

## 9. Can We Restore In Vivo IFNs Production and Susceptibility of Infected Cells?

Although significant efforts have been made for drug development to counteract SARS-CoV-2 infection, a great need for additional treatments still exists. As described before, the highly pathogenic SARS-CoV-2 uses structural and non-structural proteins to counteract the IFN system in the early and late phases of the viral life cycle. Thus, possible new targets of antiviral molecules could be viral proteins involved in counteracting the type I and III IFN system to restore a physiological production of and sensitivity to these cytokines at the site of infection and their subsequent shutdown through the numerous physiological negative feedbacks, which foresee the intervention of the ISGlase USP18 and the inhibitors of the JAK-STAT pathway (i.e., SOCS and PIAS) to avoid undesirable effects on the regulation of the immune response and tissue repair.

Due to the consistency of published data, Palermo et al. [109] suggested that Nsp1 and ORF6 could represent preferential targets of therapeutic interventions aimed at overriding the IFN blockade during the early stage of natural infection. Moreover, small-molecule inhibitors of PLpro could have a dual therapeutic effect in inhibiting the cleavage of viral polyproteins and blocking its intracellular de-ISGylation activity and other immune evasion mechanisms. Further, inhibiting PLpro would also limit the secretion and extracellular signaling of ISG15. Interestingly, it has been reported that the non-covalent inhibitor GRL-0617, which inhibits the catalytic activity of SARS-CoV-2-PLpro, could restore ISGylation of host proteins, recover IFN-signaling and decrease the number of viral particles observed in the supernatant [129]. Limiting ISG15 secretion might be expected to be detrimental in combating the infection because pro-inflammatory cytokines are essential in recruiting and activating cells of the adaptive immune response. On the other hand, if some of the most severe and deadly consequences of infection are the result of cytokine release syndrome, then limiting ISG15 secretion and signaling by therapeutically inhibiting PLpro might be beneficial [131]. As stated above, the combo of nirmatrelvir and ritonavir (Paxlovid) also targets Nsp5, but whether Paxlovid can increase IFN sensitivity, contributing to the drug effectiveness, was not explored. 

In the late phase of severe COVID-19, the induction of type I IFNs has been described to be associated with a poor outcome of the disease. A shutting down of IFNs’ sensitivity at this time through the numerous negative feedbacks could be evaluated. Several JAK/STAT inhibitors have been used to treat moderate-to-severe cases of COVID-19 to counteract the effect of the cytokine storm, which showed a significant reduction of mortality or a better clinical outcome [190,191,192]. Whether the use of a more precise inhibitor of the IFN receptors signaling late in the infection may be sufficient to hinder the overall hyper-inflammation must be better investigated.

## 10. Conclusions

Coronaviruses have evolved many strategies to contrast the IFN system, particularly the induction of type I and III production and their signaling, underlining the importance of these cytokines to contrast viral infections. During the last three years, it has become clear that an enormous heterogeneity exists in the magnitude and kinetics of the early innate immune response during SARS-CoV-2 infection, suggesting a dysregulated and/or delayed IFN response is likely associated with a poor prognosis. The multicentric analysis highlighted the association between severe COVID-19 outcomes and rare genetic variants of IFN genes and/or the presence of anti-type IFN Abs, both impairing type I IFNs signaling. Therefore, detecting congenital disabilities or auto-Abs in SARS-CoV-2 infected patients could be a prognostic factor of severe disease.

An IFN-based therapy has been considered for COVID-19 treatment. Nevertheless, while beneficial in the early phase of infection when the antiviral activity of IFNs limited SARS-CoV-2 replication, a detrimental response may be elicited in late stages, when uncontrolled IFN response could drive inflammatory lung pathology. These findings indicate that the time of an IFN-based therapy is crucial for its efficacy. In addition, the route of administration is an essential issue for possible side effects and needs to be further investigated for optimal IFNs usage. The best approach to inhibit SARS-CoV-2 replication would also avoid the selection of resistant variants, possibly targeting at the same time several viral proteins. An ideal combination therapy would therefore inhibit the viral polymerase, the maturation of the viral polyprotein precursors pp1a and pp1ab and reactivate the viral genome sensing and IFN susceptibility in the cells with a localized endogenous production of type I and III IFNs as well as the activation of the IFN-mediated innate immunity. An example of the efficacy of this type of strategy to cure viral infection come from the first eradication therapy for HCV chronic infection obtained through the combination of direct-acting antivirals (DAA) [193]. It consisted of ribavirin, directed against the viral RNAdRNAp NS5B, and an inhibitor of the viral protease NS3-4A. The NS3-4A protease is indispensable for the generation of all the viral proteins through the proteolytic maturation of the single polyprotein precursor encoded by the HCV genome. NS3-4A protease also targets the signaling intermediates of the dsRNA sensors TLR3 and RIG1 that lead to the production of IFN beta. Therefore, the efficacy of the combination therapy resulted from the inhibition of the viral polymerase and the generation of the mature viral proteins, and the local reactivation of the sensing of the viral genome that restores the IFN-mediated innate immunity.

Further studies are needed to further understand SARS-CoV-2 biology and its interference with host immune responses. They will provide a more profound knowledge of the processes and mechanisms involved in immune-mediated viral clearance and immune-evasion strategies and better strategies for the treatment of COVID-19 or other emergent viral infections.

## Data Availability

Not applicable.
